# Background choice and immobility as context dependent tadpole responses to perceived predation risk

**DOI:** 10.1038/s41598-020-70274-w

**Published:** 2020-08-11

**Authors:** Paula Cabral Eterovick, Jéssica Stephanie Kloh, Cleber Cunha Figueredo, Pedro Igor Macário Viana, Marcella Goulart, David Travassos Milan, Melissa Bravo Fonseca, Ítalo Moreira Martins, Luan Tavares Pinheiro, Rúbia Praxedes Quintão, Thais Kelly Fagundes Melo, Rodolfo Assis Magalhães, Caio Motta Campos, Vanessa Cristina Monteiro Ferreira, Ana Laura de Oliveira, Miguel Vences

**Affiliations:** 1 Programa de Pós-Graduação em Biologia de Vertebrados, Pontifícia Universidade Católica de Minas Gerais, Belo Horizonte, Minas Gerais 30535-610 Brazil; 2 Programa de Pós Graduação em Ecologia, Conservação e Manejo de Vida Silvestre, Universidade Federal de Minas Gerais, Belo Horizonte, Minas Gerais Brazil; 3 Programa de Pós-Graduação em Ecologia, Universidade Federal de Viçosa, Viçosa, Brazil; 4 Zoological Institute, Braunschweig University of Technology, Spielmannstr. 8, 38106 Brunswick, Germany

**Keywords:** Behavioural ecology, Evolutionary ecology

## Abstract

The association of immobility and camouflage is widespread as a defensive mechanism in prey from varied taxa. However, many experiments assessing the reaction of prey to predator cues are conducted under artificial laboratory conditions. In a previous experiment we observed the tadpoles of *Ololygon machadoi* (Hylidae) to respond to predator visual and/or chemical cues by choosing backgrounds that improve their disruptive properties, but detected no associated reduction of movement. Here we experimentally demonstrate this response in the species' natural habitat, on backgrounds where the tadpoles are likely to achieve their best camouflage. We also tested whether previous experiences could influence both background choice and immobility in *O. machadoi* tadpoles. These novel experimental results suggest that a defensive behavior—i.e., reduction of movement—in these tadpoles is more strongly expressed under the natural conditions where they evolved, compared to laboratory conditions where prey and predator were brought into closer contact. Besides, previous experiences are likely to play an important role in expressed defensive responses.

## Introduction

Prey species are under constant selection to escape predation. They can develop predator specific responses, but some responses seem to be widespread, such as spatial avoidance of predators and reduction of activity^1^. In this context prey species that count on camouflage to reduce the probability of being detected by predators tend to remain motionless, aiding to the protective properties of their colouration^2,3^. Reduction of movement is likely to reduce detection probability and can emerge as a spontaneous response when the detected threat is at a distance (background threat), but an approaching (immediate) threat may otherwise elicit an escape response^4^. Previous experience can also determine the reaction of prey to specific predators^5^, however there is evidence that evolutionary pressures may have led prey species to develop different defensive mechanisms and express each one in appropriate contexts determined by the assessed threat^4^. Both innate and learned defensive behaviours can be expressed even in very early life stages^6,7^.

Tadpoles, the larvae of biphasic anuran amphibians, are frequently employed in experiments conducted to study the reaction of prey to predator cues^8^, due to their broad range of predators and defensive mechanisms^1^. It has been shown that many species of tadpoles reduce movement in the presence of predators^1,9^. However, these studies are usually conducted under laboratory or mesocosm conditions, sometimes within reduced spaces in artificial standardized containers where predator cues are manipulated, other factors kept equal, to record tadpole reaction to particular cues^1,10^. Under such circumstances, both visual and chemical stimuli have been shown to elicit anti-predator responses in tadpoles^11,12^. In natural habitats, on the other hand, tadpoles should be selected to identify and react to predator cues within a more complex context of stimuli, representing a more realistic situation.

Although tadpole neurons may have little sensitivity in early life stages, they can quickly evolve to deliver sensory-driven spikes to the developing brain when exposed to natural visual stimuli, such plasticity being adaptive to the animal’s sensory environment^13,14^. *Xenopus* tadpoles can respond to visual stimuli as soon as retinal ganglion cells innervate in the brain, different from other vertebrates, who develop this response days or even weeks later, what may be an adaptation to the great need to detect and avoid predators^15^. On the other hand, the visual acuity of tadpoles may at least in some cases not be sharp enough to detect predators visually at distances beyond their close vicinities, as studies conducted on some species show that they are myopic^16^. However, as the aforementioned studies were conducted on single species, the data available is still too limited to take conclusions about the accuracy of tadpole vision in general.

The treefrog *Ololygon machadoi* has conspicuously coloured larvae: especially the young tadpoles have a dark brown body with an anterior and a posterior transversal yellow band, and this pattern is expected to be disruptive on yellowish backgrounds^17^. In laboratory experiments, these tadpoles increased the use of yellow backgrounds in the presence of visual and/or chemical cues of water bugs (*Belostoma testaceopallidum* Latreille, 1807)^18^, but did not show decreased movement as would be expected in association with background matching^3^. In the Melo et al.^18^ study, visual, chemical and both stimuli were provided by addition of a water bug inside a transparent container, a black container with holes, and a transparent container with holes, respectively, to the trays where tadpoles were tested. The tadpoles used in that study were collected at the same location of the present study and were immediately taken to the laboratory inside plastic bags with water from the original stream in polystirene foam boxes. At the laboratory, they were kept under natural light, in the shade, and tested within the next 10 days.

Although laboratory experiments can be carefully planed to avoid stress to the experimental subjects, they increase precision in detriment of realistic conditions^19^. The lack of a decreased movement response of the *O. machadoi* tadpoles observed under such experimental conditions may have been due to the proximity of the predator eliciting an escape response. Otherwise, the effects of previous experiences may have induced varied individual reactions, masking the effects of tested predator cues. Thus, we decided to replicate this experiment in situ, in the natural habitat where the tadpoles occur and using the same native predators, in order to check whether tadpoles would reduce their movement rate under these more realistic conditions when exposed to predator cues at a greater distance.

In order to evaluate whether previous experience can influence expression of defensive behaviours, we also tested whether tadpoles could perceive backgrounds as safer and increase their use in response to previous experiences. Because movement may be important to elicit defensive responses^14^, we tested whether tadpoles of *O. machadoi* would respond differently when subject to aversive stimuli (an approaching object) on different backgrounds by using an alternative (“safer”) background. We also tested for decreased mobility after exposure to such aversive stimuli. In this case, because the needed background manipulation could not be done in situ, we conducted the experiments in trays with pictures of natural backgrounds on the bottom, with stream water and natural light and temperature conditions at the study site.

## Results

**Figure. 1:**
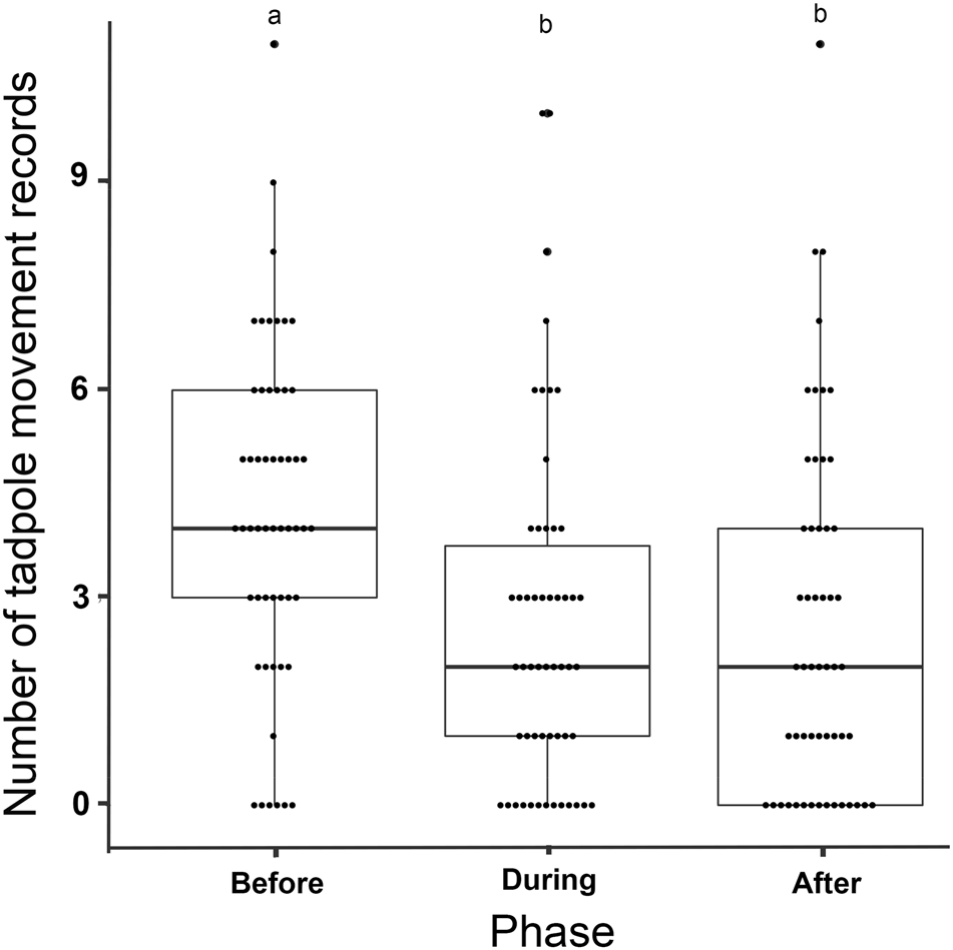
Activity levels (number of recorded movements—see methods for details) of *Ololygon machadoi* tadpoles before, during, and after exposition to visual and chemical cues of *Belostoma testaceopallidum*. The original data is shown in dot plots with box plots representing means and the limits of 1st and 3rd quartiles. Results that differed significantly are indicated by different letters.

Tadpoles of *Ololygon machadoi* reduced their movement in the presence of predator cues in their natural habitat. The best model included only phase (before, during, or after the presence of the water bug) as explanatory variable to tadpole level of activity (*p* < 0.0001; Table 1). Weather had no influence on tadpole behaviour regarding number of movements, as the model including only weather had lower explanatory power than the null model (Table 1). Tukey post hoc tests indicated that tadpoles moved significantly more before predator presentation than both during predator presence (estimate = 0.371, SD = 0.093, z = 4.000, *p* = 0.0002) and after predator removal (estimate = 0.376, SD = 0.093, z = 4.050, *p* = 0.0002; Fig. 1). However, number of movements did not differ during predator presence and after its removal (estimate = 0.005, SD = 0.100, z = 0.051, *p* = 0.998; Fig. 1). Although we did not monitor tadpole position in the enclosures, we noticed no tendency of tadpoles to move away from the predator.

**Table 1:** Models built to (A) evaluate the level of activity of tadpoles of *Ololygon machadoi* (Hylidae) (given by the number of instant positive records of movement) before, during, and after the manipulated proximity of a predator (*Belostoma testaceopallidum*, Belostomatidae) in experimental enclosures and (B) assess the effect of previous aversive experiences in background avoidance and movement reduction. Values of AIC and AICc are presented and the selected model is presented in boldface.

	AIC	AICc
A. Models to explain number of tadpole movements		
**nmovements ~ phase + (1|tadpole)**	**719.21**	**719.46**
nmovements ~ phase + weather + (1|tadpole)	721.05	721.43
nmovements ~ phase*weather + (1|tadpole)	723.91	721.43
nmovements ~ weather + (1|tadpole)	739.15	-
nmovements ~ 1 + (1|tadpole) [null model]	737.30	-
B. Models to evaluate the effect of previous experience on tadpole background choice and level of movement		
darkbackground ~ phase*treatment + (1|tadpole)	**1,041.00**	**1,041.65**
darkbackground ~ treatment + (1|tadpole)	1,043.56	1,043.79
darkbackground ~ 1 + (1|tadpole) [null model]	1,044.84	1,044.91
movements ~ phase*treatment + (1|tadpole)	**1,126.23**	**1,126.88**
movements ~ phase + treatment + (1|tadpole)	1,127.29	1,127.64

For the experiment conducted to evaluate the effect of previous experience on background choice by tadpoles, the best model included phase, treatment and their interaction (*p* = 0.017, Table 1, Fig. 1). Dark background usage frequency differed significantly between the two treatments after the aversive stimulus was applied: tadpoles used significantly more the dark backgrounds after they experienced aversive stimuli on the yellow background than on the dark background itself (Tukey estimate =  − 0.248, z =  − 3.40, *p* = 0.009). As expected, no differences were observed within the control and among the control and the phases before the aversive stimuli in the treatments.

**Figure. 2:**
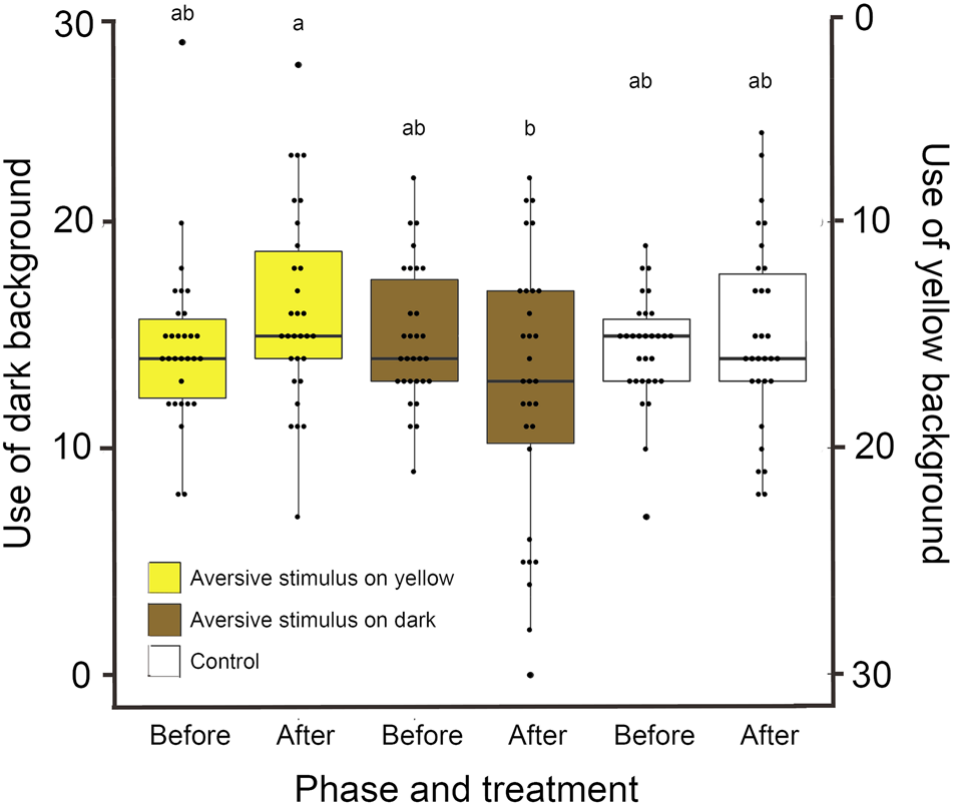
Usage frequency of dark and yellow backgrounds by *Ololygon machadoi* tadpoles after exposed to aversive stimuli on yellow backgrounds, on dark backgrounds or receiving no aversive stimuli. The original data is shown in dot plots with box plots representing means and the limits of 1st and 3rd quartiles. Treatments with aversive stimuli are represented in the colours where the stimuli were applied (that tadpoles were expected to associate with danger). Results that differed significantly are indicated by different letters.

For tadpole activity, the best model also included phase, treatment and their interaction (*p* < 0.001, Table 1, Fig. 2). Tadpoles moved less after the treatments with aversive stimuli were applied on the dark backgrounds (Tukey estimate =  − 0.299, z =  − 4.234, *p* < 0.001) but no changes in activity levels happened in the control (Tukey estimate =  − 0.11391, z =  − 1.724, *p* = 0.505) or in the treatment with aversive stimuli on the yellow background (Tukey estimate =  − 0.10114, Z =  − 1.477, *p* = 0.669; Fig. 2).

## Discussion

In a previous study under laboratory conditions^18^, no reduction of activity levels was observed for *Ololygon machadoi* tadpoles in response to visual and / or chemical cues from water bugs (*Belostoma testaceopallidum*). On the contrary, here we show that the tadpoles reduce their activity levels in situ when confronted with predators at considerable distances (up to 35 cm). They also maintained the reduced mobility response for the next 15 min phase of the experiment. This is in accordance with observations on *Xenopus laevis* tadpoles, that maintained a learned danger avoidance response even after 180 min of an experienced aversive stimulus^20^.

Tadpoles are likely selected to detect their predators before they are detected^10^, and this may explain the ability of tadpoles to respond to the predator under natural conditions where predator cues are less noticeable. Our results suggest that the artificial conditions of laboratory experiments may sometimes alter animal behaviours, so extrapolation of results to in situ conditions should be done carefully. It is possible that tadpoles assessed their natural backgrounds as safer (regarding camouflaging properties) than the artificial backgrounds used in laboratory experiments and thus expressed their associated immobility behaviour more efficiently. It is also possible that tadpoles did not respond to predator cues properly under laboratory conditions due to additional stress caused by the unfamiliar artificial surroundings.

An alternative explanation for our results could be a higher initial movement rate to explore new surroundings followed by tadpole settling down / getting tired and moving less. Although we did not test for this possibility in the in situ experiment, it seems unlikely based on the results of the experiment conducted in the trays. Tadpoles were placed in trays with a similar area (1,255 cm^2^ compared to 1,290 cm^2^ in the in situ experiment) and in the controls, where tadpoles received no aversive stimuli, as well as in treatments aimed at associating the yellow background with threat, movement rates did not change over time. These experiments lasted 105 min, a time period longer than the duration of the experiments conducted in the stream (54 min) and still not enough for tadpoles to decrease movement rates without interference (an aversive stimulus or predator).

**Figure. 3:**
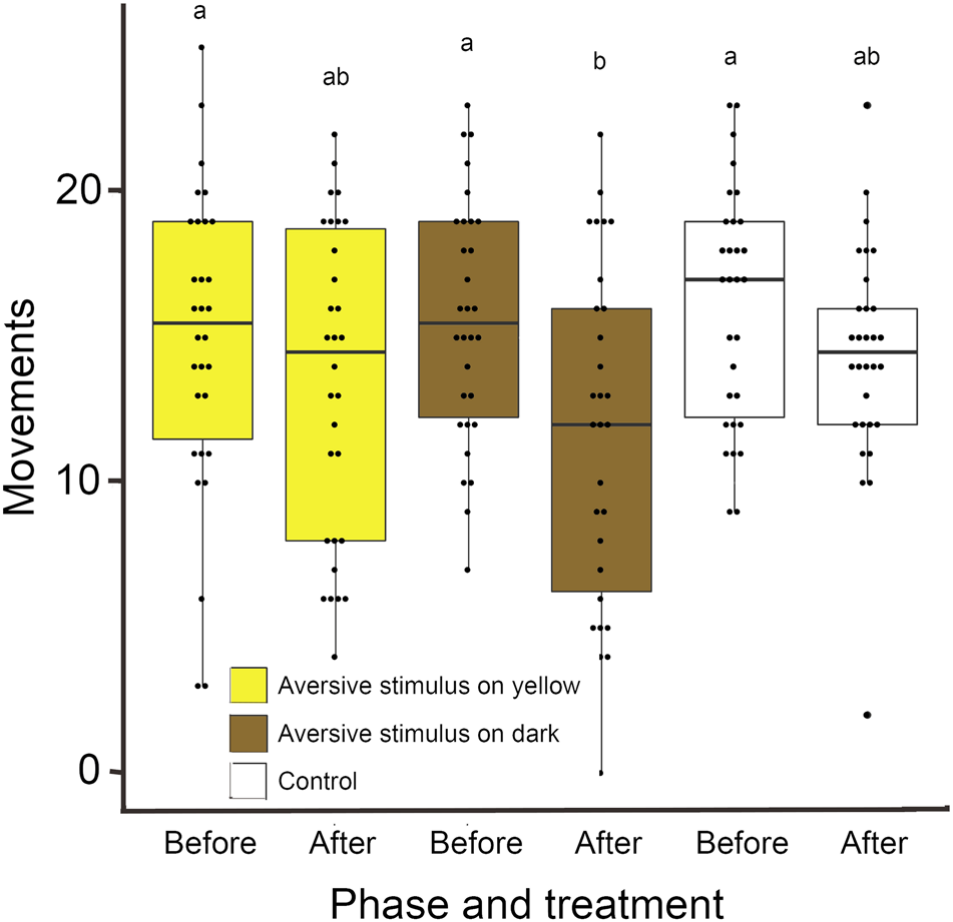
Background change frequency (as a surrogate for activity) by *Ololygon machadoi* tadpoles after exposed to aversive stimuli on yellow backgrounds, on dark backgrounds or receiving no aversive stimuli. The original data is shown in dot plots with box plots representing means and the limits of 1st and 3rd quartiles. Treatments with aversive stimuli are represented in the colours where the stimuli were applied (that tadpoles were expected to associate with danger). Results that differed significantly are indicated by different letters.

Our results show that tadpoles of *Ololygon machadoi* were able to learn to avoid backgrounds where they are in danger based on colour. The ability to associate aversive stimuli with specific habitats and avoid them may be an important adaptation to avoid predation^21^. *Xenopus laevis* tadpoles learned to avoid specific wavelengths and intensities associated to aversive stimuli under laboratory conditions^20^, indicating that tadpoles can recognize colour and learn from their experiences. *Ololygon machadoi* tadpoles also showed a significantly decreased movement response when presented to aversive stimuli on dark backgrounds and moved towards yellow backgrounds (see Fig. 3), what may reinforce the protective effect of camouflage. Innate preferences are likely to influence on the results of learning processes^20^, and an innate (or learned) preference for yellow backgrounds seems to prevail in tadpoles of *O. machadoi*^17^. When moving towards yellow backgrounds, the tadpoles may be more likely to express immobility if they have an innate or learned ability to associate yellow backgrounds with protection (through camouflage).

Reduction of movement rates is a very widespread defensive mechanism among tadpoles^1,22,23^, and the association between camouflage and immobility for defense occurs not only in tadpoles, but also in adult frogs^2^ and other animals^3^. Movement is likely to undo the protective effect of camouflage, so that the effects of camouflage and immobility are synergistic in reducing detection rates and, expectedly, predation^2,3^. The widespread association of camouflage and immobility in varied prey species corroborates the selective value of the movement decrease response for prey, what may explain why tadpoles were able to react to predator presence in the natural habitat, even with likely subtle cues and other potentially influencing variables (e.g. food availability). In the natural habitat they were exposed to the trade-off between moving to eat and standing still in the presence of food (that was not available during the experiments in the laboratory), so they would have an additional reason to move^24^. The reduction of movement in the presence of the predator (and shortly after it) reinforces the adaptive importance of this behaviour.

In summary, our results show that tadpoles of *Ololygon machadoi* can adjust their defensive behaviour based on surrounding conditions and previous experiences, fine-tuning their anti-predator strategies. Similar abilities have also been shown for other species^25^. This flexibility should be taken into account in studies on tadpole behaviour in order to best adjust experimental design to aims and to interpret results with care.

## Methods

### Study site

Experiments were conducted in the Reserva Particular do Patrimônio Natural (RPPN) Santuário do Caraça, a private conservation unit in the southern portion of the Espinhaço Mountain range, Minas Gerais state, Brazil. The climate is seasonal, with a rainy period from October to March, and a dry period from April to September. Mean air temperatures vary between 13 and 29^o^C^26^.

The focal species of this study was *Ololygon machadoi.* The tadpoles of this treefrog have been previously shown to react to both visual and chemical predator cues (from *Belostoma testaceopallidum*; Melo et al.^18^) by positioning themselves preferentially on yellow backgrounds where they are disruptive^17,18^. *Ololygon machadoi* breeds year-round in many streams in the RPPN, and we used one of these streams (20^o^05′37″S, 43^o^29′59″W; 1293 m above sea level), where its tadpoles are abundant, to conduct the experiments. It is a small first order stream^27^ with sandy or rocky bottoms. Stream bank vegetation is dense, composed of herbs, shrubs and trees. In the vicinities of the point where we conducted the experiments (and up to 150 m upstream) stream width ranges from 2 to 6 m, and stream depth, from just a few centimeters to about 1 m^28^.

### Response to predator cues in the natural habitat

**Figure. 4:**
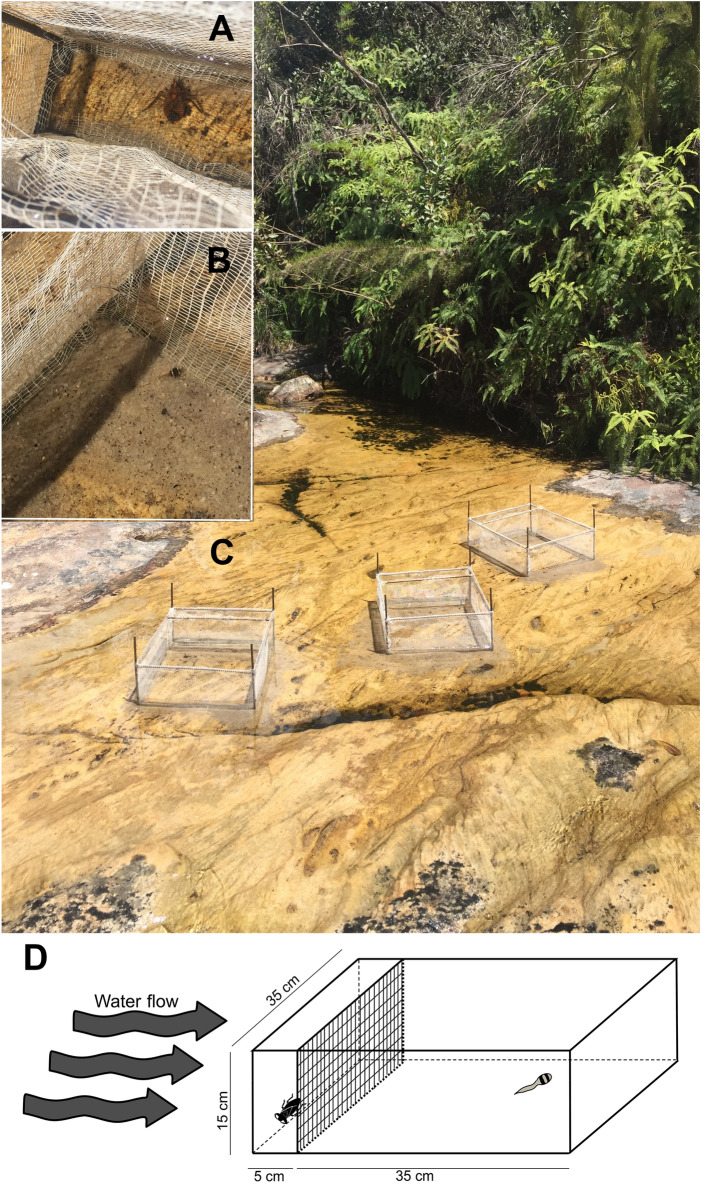
Experimental design showing the water bug predators (*Belostoma testaceopallidum*, (**A**) and tadpoles of *Ololygon machadoi* (**B**) inside enclosures placed in a stream (**C**) at the RPPN Santuário do Caraça, Southeastern Brazil, where both species occur. A schematic representation of the experimental enclosures is also shown (**D**).

We built three enclosures measuring 40 × 35 cm with a plastic mesh around a metal frame that limited two compartments, one measuring 35 × 35 cm, and another contiguous to it measuring 5 × 35 cm. Both compartments were 15 cm high and were open both below and above. We set the enclosures at a stream section of shallow water and flat rocky bottom, where water filled the enclosures up to about 5 cm (Fig. 4). We placed the enclosures with the small compartment upstream, so that the tadpole in the larger compartment would be exposed to both visual and chemical cues of the predator to be introduced in the smaller compartment. We collected three water bugs (*Belostoma testaceopallidum*) to be used in the experiments in a nearby stream from the same water basin (20^o^06′40″S, 43^o^28′48″W; 1,254 m above sea level). We collected the tadpoles very close to the enclosures (up to 5 m upstream).

Before we started each trial, we inspected the area covered by the enclosures to make sure no tadpoles or other animals remained inside. We then sealed the bottom carefully with sand from the same stream and placed one tadpole within each cage, in the larger compartment. We waited 3 min, sufficient for the tadpole to return to normal activity levels after translocation to the enclosures^17^, and then we recorded whether each tadpole was moving or standing still in 30 s intervals, during 15 min for a total of 30 observations. When tadpoles moved, they always moved on the bottom, never through the water column. After this, we waited another 3 min and repeated the 30 movement records for the next 15 min. We then removed the water bug and repeated another observation turn (waited 3 min, then made 30 movement observations separated by 30 s intervals). After each individual tadpole was tested, we released it downstream, to avoid using the same individual more than once. Tadpoles were all in developmental stage 25^29^ and measured 20.2 ± 2.4 mm (n = 33 tadpoles measured). We maintained the water bugs in individual recipients with clean stream water and used them randomly in the three enclosures. After all the experiments we collected them for identification.

We tested 3 tadpoles simultaneously, then restarted the whole experiment with other 3 tadpoles, and so on, until we tested 54 tadpoles during three consecutive days (3–5 October, 2018). The weather was sunny with some clouds and short periods of light rain, during which we did not conduct experiments.

### Defensive responses based on previous experiences

For this experiment, we collected stage 25 tadpoles at the same stream section and kept them for no more than 2 h in polystyrene boxes with stream water by the nearby (about 1.3 km) lodging of the RPPN Santuário do Caraça, where we conducted the experiments to test the influence of previous experience on tadpole background choice and immobility. We placed individual tadpoles in plastic trays measuring 43 cm length, 30 cm width, 9 cm height, with half the bottom covered by a picture of a natural yellow background (rocks in its natural habitat). The other half was covered with the same picture manipulated digitally to match the hues and luminance of natural dark backgrounds, as in^17^. We filled the trays with tap water that comes straight from the main stream at the reserve, replacing the water at every trial. For each trial, we waited 3 min. after tadpole placement in the center of the tray, then we observed tadpoles for 30 min, recording their background every minute. After that, we applied one of three treatments during a 5-min interval: (1) an aversive stimulus was applied to the tadpole every time it positioned itself on the yellow background, or (2) on the dark background or (3) no stimulus was applied (control). The aversive stimulus consisted in one person approaching a wood stick slowly towards the tadpole until it reacted fleeing. After the treatments, we conducted another 30 min of observations recording tadpole background every minute. The tadpoles were all returned to their original stream after the experiments. We tested 2 tadpoles in each treatment simultaneously and then repeated the trials until tests of 30 tadpoles for each treatment (total 90 tadpoles) were completed. Experiments were performed from 4 to 6 October 2016. Experiments were conducted in the shade with natural light, and all days were sunny.

All the procedures were performed in accordance with relevant guidelines/regulations adopted by the responsible institutions: Sisbio/ICMBio (45302-1, 62316-1) authorized animal manipulation in situ and the Ethical Committee of the Pontifícia Universidade Católica de Minas Gerais (032/2016, 003/2018) approved the experimental procedures in accordance with animal welfare guidelines. The water bugs were identified as *Belostoma testaceopallidum* Latreille, 1807, and the collected specimens were deposited in the collection of aquatic insects of the Parasitology Department of the Institute of Biological Sciences (DPIC) of the Federal University of Minas Gerais, Belo Horizonte, Minas Gerais state, Brazil, under the accession number 9498.

### Statistical analyses

We compared the level of activity of tadpoles (given by the number of instant positive records of movement) before, during, and after the presence of the water bug in the enclosures in the stream. We also evaluated a possible effect of direct sun incidence or shade on the enclosures^30^, and its interaction with tadpole activity levels. We built Generalized Linear Mixed Models (GLMM) with the packages “car”^31^ and “MASS”^32^ in R^33^. We considered number of movement records + 1 (to adjust to distributions that must be non-zero) as the dependent variable, phase (before, during, or after the presence of the water bug) and light (sun or shade) as explanatory variables, and individual tadpole as a random variable. Tadpoles might present different reactions to predators based on their previous experiences^34^. Considering individual as a random variable would also account for possible differences among times of the day and cages on individual behaviour. We built models including each one or both explanatory variables, with or without their interaction. We compared these models with a null model that included only the random variable, in order to identify the variable(s) with the strongest explanatory power.

We also used GLMMs to test for the ability of tadpoles to avoid a background colour after an aversive experience on it. Since tadpoles had to choose between dark and yellow, we arbitrarily used the number of records on dark backgrounds as dependent variable, because the records on yellow would represent the alternative situation (not on dark). We used treatment and phase (before and after the treatments were applied) as fixed variables and tadpole as a random variable. In order to test for the expression of immobility after the aversive stimuli, we considered the number of times tadpoles changed background colour in consecutive observations as a surrogate for tadpole movement (our dependent variable), treatment and phase as fixed variables and tadpole as a random variable.

We used the package MuMIn^35^ for R^33^ to select the best models, a procedure recommended to control the overall type I error rate^36^. We conducted Tukey post hoc tests with the package emmeans^37^.
